# TopUp SERS Substrates with Integrated Internal Standard

**DOI:** 10.3390/ma11020325

**Published:** 2018-02-24

**Authors:** Sophie Patze, Uwe Huebner, Karina Weber, Dana Cialla-May, Juergen Popp

**Affiliations:** 1 Leibniz Institute of Photonic Technology e. V. (IPHT), Albert-Einstein-Str. 9, 07745 Jena, Germany; patzesophie@gmail.com (S.P.); uwe.huebner@leibniz-ipht.de (U.H.); karina.weber@leibniz-ipht.de (K.W.); 2 Institute of Physical Chemistry and Abbe Center of Photonics, Friedrich-Schiller-University Jena, Helmholtzweg 4, 07743 Jena, Germany

**Keywords:** SERS, TopUp SERS substrate, sulfamethoxazole, Langmuir–Freundlich isotherm

## Abstract

Surface-enhanced Raman spectroscopy (SERS) is known as a molecular-specific and highly sensitive method. In order to enable the routine application of SERS, powerful SERS substrates are of great importance. Within this manuscript, a TopUp SERS substrate is introduced which is fabricated by a top-down process based on microstructuring as well as a bottom-up generation of silver nanostructures. The Raman signal of the support material acts as an internal standard in order to improve the quantification capabilities. The analyte molecule coverage of sulfamethoxazole on the surface of the nanostructures is characterized by the SERS signal evolution fitted by a Langmuir–Freundlich isotherm.

## 1. Introduction

Surface-enhanced Raman spectroscopy (SERS) combines the fingerprint specificity of Raman spectroscopy with potential single-molecule sensitivity due to the interaction of molecules with plasmonic metal nanostructures [1,2,3,4]. Thus, analyte molecules can be detected achieving molecular information in trace levels using SERS-based detection schemes. A broad variety of application fields in bioanalytics and biomedicine are known for SERS and have been well summarized by recently published review articles [5,6,7]. However, despite this huge potential of SERS in (bio)analytics, the technique is not accepted in routine analytics, which is often associated with the lack of reproducibility of the applied SERS substrates. To address this obstacle, a number of fabrication strategies are available claiming the preparation of ‘reproducible SERS substrate’ [8,9,10,11,12,13,14]. Additionally, SERS is combined with microfluidic approaches in order to allow for comparable and reproducible measuring conditions in quantitative detection schemes [15,16,17]. 

In order to address variations in laser intensity, focus, and setup alignment, an internal standard for signal intensity normalization is necessary for reliable quantitative results [18]. For SERS, special demands on the standard method are needed, since not only the aforementioned issues have to be addressed but also the fluctuating enhancement of the SERS signal, resulting from the heterogeneous distribution of the local electromagnetic field across the nanostructured surface. Furthermore, the Raman signal of the internal standard has to be detectable without suppressing the SERS signal of the molecule of interest. Within the literature, different standardizing strategies are introduced and discussed. As an example, for the detection of pharmaceuticals in tablets based on their Raman signal, the tablet matrix was used as an internal standard [18]. In the case of SERS, the application of the Raman signal of the matrix molecules as an internal standard might be disadvantageous due to the competition for binding sites on the metallic surface [19]. Therefore, substances, such as thiocyanate with a marker mode around 2200 cm^−1^ (C≡N stretching vibration), which have SERS signals in the silent spectral region are applied as an internal standard [20].

To address local fluctuations in SERS intensity, molecules competing with the analyte for free binding sites on the nanoparticle surface are used. A prominent standardizing method is the addition of isotopes to the investigated sample [21], which show the same chemical behavior as the analyte molecule and, therefore, the same signal enhancement properties are expected. The drawback of this method is that the two substances compete for free binding sites on the nanostructured surface and their Raman marker modes might overlap. In another study where colloidal nanoparticles were used, the internal standard was incorporated within the metallic multilayer. In this way, a reasonable Raman signal intensity of the internal standard could be obtained without blocking the binding sites on the external surface of the particles [22]. As an example, a gold core is modified with a Raman reporter layer, e.g., aminothiophenol, and later covered by a silver shell which acts as the enhancing material for the analyte molecules. 

In the case of SERS substrates with high structural reproducibility across the surface, less SERS signal fluctuations are expected. Additionally, by averaging the SERS signal across the nanostructured surface, small fluctuations are levelled out. Thus, an internal standard to correct on the local SERS intensity fluctuations is not needed. Using the example of planar substrates fabricated by nanosphere lithography (NSL), the polystyrene signal originating from the preparation process [23] or the silicon from the substrate basis [24] can be utilized as an internal standard to address variations in laser intensity, focus, and setup alignment. 

Within this manuscript, we introduce the application of silicon as an internal standard for our recently developed TopUp substrates [25]. Here, silicon is used as supporting material for the SERS active substrate. This TopUp substrate successfully combines the benefits of a top-down structure with the fast and easy preparation of a bottom-up crystalline silver nanostructure. Moreover, the SERS signal distribution is highly homogenous, allowing for quantitative measurements. Due to the presence of silicon as supporting material, both the SERS signal from the analyte molecule and the Raman signal of silicon are recorded simultaneously. The antibiotic sulfamethoxazole is applied for all investigations and the benefits brought by the internal standard are illustrated by the extended linear dynamic range. Finally, it is found that the SERS intensity profile follows a Langmuir–Freundlich isotherm.

## 2. Results and Discussion

**Figure 1:**
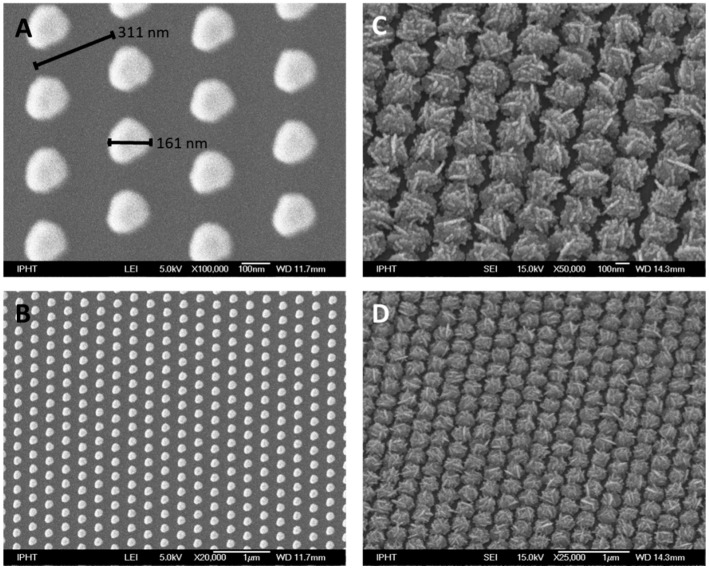
SEM images of the gold island template structure (**A**,**B**) and the corresponding TopUp silver structure after self-organized silver deposition (**C**,**D**).

SEM images of the gold template as well as of the created TopUp@silicon substrates are illustrated in Figure 1. The gold island film consists of hexagonally arranged gold dots on a silicon wafer as shown in Figure 1A. The dot size is in the range of 160 nm and the period is ~310 nm. This nanostructured surface was achieved by a combined electron beam lithography and lift-off process as mentioned within chapter 4 ‘Materials and Methods’. Thus, the isolated gold islands serve as seeding particles for the bottom-up synthesis of silver nanostructures [25]. The arrangement of the gold islands template structure was chosen as a closest packaging of hexagonal arranged dots in order to allow for high SERS signal intensities and homogenous distribution. Thus, a high coverage of silver nanostructures on the surface is guaranteed. The SEM images of the resulting TopUp@silicon substrate are depicted in Figure 1C,D, illustrating that the arrangement of the gold islands is preserved on the final SERS active substrate. As shown, homogeneously and perfectly ordered silver nanostructures with interstitial silicon regions were fabricated. Thus, the Raman signal of silicon will serve as an internal standard since it is expected that both the SERS signal of the analyte molecule as well as the Raman signal of the supporting material will contribute to the overall readout signal.

**Figure 2:**
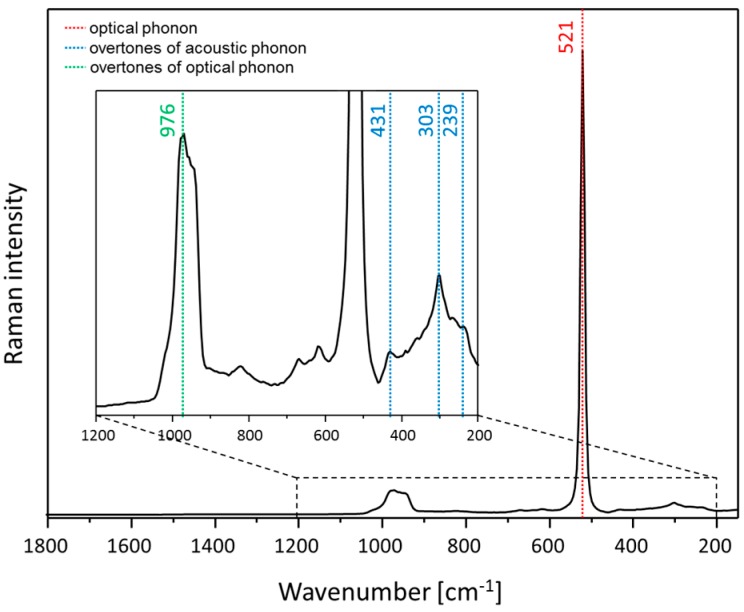
Raman spectrum of crystalline silicon. The Raman spectrum is dominated by the optical phonon mode at 521 cm^−1^. Overtones of optical phonons as well as acoustic phonons are marked accordingly.

Silicon is a semiconductor material which shows two prominent bands in the Raman spectrum [26], as shown in Figure 2: one very intense peak based on optical phonons at 521 cm^−1^ and one broader and less intense peak assigned to overtones of optical phonons at 960 cm^−1^. A more detailed description of the silicon Raman spectrum can be found elsewhere [27,28,29]. It is reported in the literature that an excitation in the lattice vibration of the *z*-plane based on laser irradiation is not possible since lasers are typically polarized in the *xy*-plane. Nevertheless, due to the focusing of the laser light through an objective and the radiation of the enhanced field around silver nanoparticles in all spatial directions, *z*-polarized phonons of silicon can be excited [27]. Therefore, the silicon signal can be enhanced; however, due to the evanescent character of the enhanced field around the silver nanoparticle, the silicon layer experiences an enhanced field only for a few nanometers in depth. Thus, the weak SERS signal of the silicon will be overlapped by the Raman signal of the bulk silicon material. Since the marker band of silicon at 521 cm^−1^ is very intense and sharp it will be used as an internal standard for our SERS investigations by using the TopUp@silicon structures as SERS substrates. By implementing the internal standard in the substrate, differences induced by the optical pathway alignment (e.g., laser intensity and focus variation) are corrected. However, variations in SERS intensity due to local differences in the signal enhancement will not be accounted for. In a previous study, the potential of the TopUp SERS substrates for quantitative analysis was already introduced [25], i.e., the reliable detection of the analyte sulfamethoxazole in the nanomolar range is achieved by a homogenous signal distribution across the surface. Moreover, due to the applied measuring conditions (scanning mode), the SERS signal intensity is averaged across the surface to minimize fluctuations in the recorded SERS signal due to local changes in the plasmonic behavior. Thus, it is guaranteed that the SERS spectra are measured with a high comparability.

**Figure 3:**
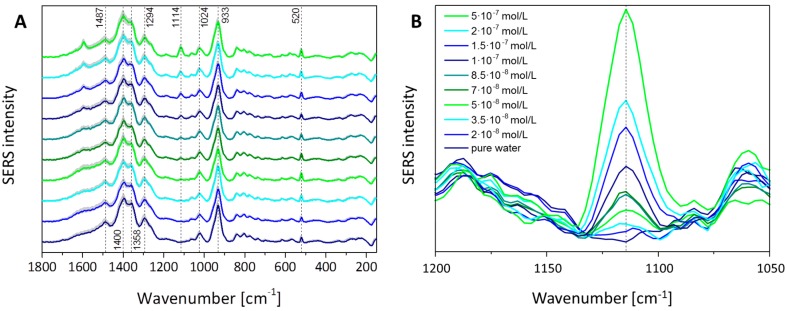
Concentration-dependent surface-enhanced Raman spectroscopy (SERS) signal intensity of sulfamethoxazole (SMX): (**A**) The marker mode of SMX at 1114 cm^−1^ is marked and in (**B**) the enlarged Figure section is shown, illustrating the increase in signal intensity with increasing concentration.

To prove the sensitivity of the TopUp@silicon substrate as well as the potential for water analytical detection schemes, the limit of detection (LOD) for the antibiotic sulfamethoxazole (SMX) in aqueous solution was investigated. Therefore, the SERS signal of a concentration row of the antibiotic SMX was detected. Due to the environmental relevant detection limit of 2 × 10^−7^ M in surface water [30,31,32], the SERS investigations were performed within the concentration range between 2 × 10^−8^ and 5 × 10^−7^ M, while using one TopUp@silicon substrate per measured concentration. The SERS spectra are depicted in Figure 3A. Since the fabrication of the silver nanostructures is realized by using hydroquinone in citric buffer as the reducing agent, hydroquinone, citrate, and also the redox reaction product benzoquinone can interact with the freshly prepared silver surface. Thus, these molecules dominate the SERS response, especially in the case of low-concentration analyte solutions since the displacement of the stabilizing analyte molecules by SMX might not be as efficient as for higher concentrations. As a consequence, the SERS spectra depicted in Figure 3A show a contribution from the redox reactants present during the fabrication process, as illustrated by the depicted SERS spectrum in the case of pure water as reference. The mode at 521 cm^−1^ is assigned to the optical phonon of silicon (see Figure 2) and its peak area is used to normalize the peak area values of the marker mode of SMX. Moreover, vibrational modes are assigned to citrate, i.e., its vibrational mode νs(COO) is located at 1400 cm^−1^; its mode δ(COO) is around 1294 cm^−1^; and the peaks at 1024 cm^−1^ and 933 cm^−1^ are due to ν(C–O) and ν(C–COO), respectively [33]. The Raman mode at 1487 cm^−1^ is assigned to the redox reactant hydroquinone and the mode at 1358 cm^−1^ is related with the vibrational mode β(CH) of the redox product benzoquinone [34]. Finally, the marker Raman mode of SMX at 1114 cm^−1^ assigned to the vibrational mode of the sulfonyl group [35] is clearly identified and the enlarged image section of the relevant wavenumber range, depicted in Figure 3B, illustrates an increase of the marker mode with an increase of the analyte concentration. The slight shift to lower wavenumbers with increasing concentration is associated with the interaction between the adsorbed monolayer of SMX molecules with molecules from further layers [35]. The background measurement in pure water shows no signal in this wavenumber region, whereas for the lowest measured concentration of 2 × 10^−8^ M, the marker band occurs and increases with higher concentration.

**Figure 4:**
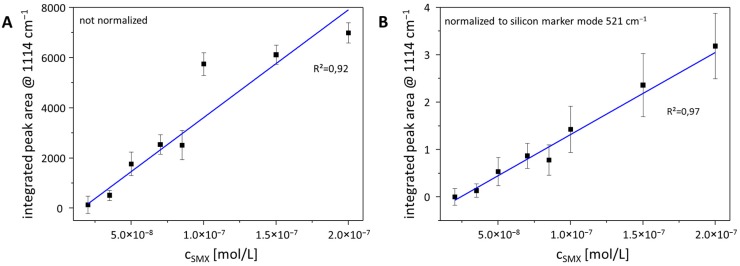
Concentration-dependent peak area as function of the SMX concentration (**A**) without normalization and (**B**) after normalization by using the peak area of the silicon marker mode at 521 cm^−1^ as internal standard.

In order to illustrate the potential for quantification in the sub-µM range reflecting the environmentally relevant concentration region, the peak area of the SMX marker mode at 1114 cm^−1^ was estimated by using the Simpson rule. In Figure 4A, the signal evolution within the range of 2 × 10^−8^ and 2 × 10^−7^ M is depicted without any normalization procedure. The correlation efficient for a linear fit is 0.92. This value is increased up to 0.97 in the case of normalization of all data on the peak area of silicon at 521 cm^−1^, as shown in Figure 4B. Thus, by using the peak area of the marker mode of silicon as internal standard, the quantification potential is increased, providing a powerful procedure for environmental analysis.

**Figure 5:**
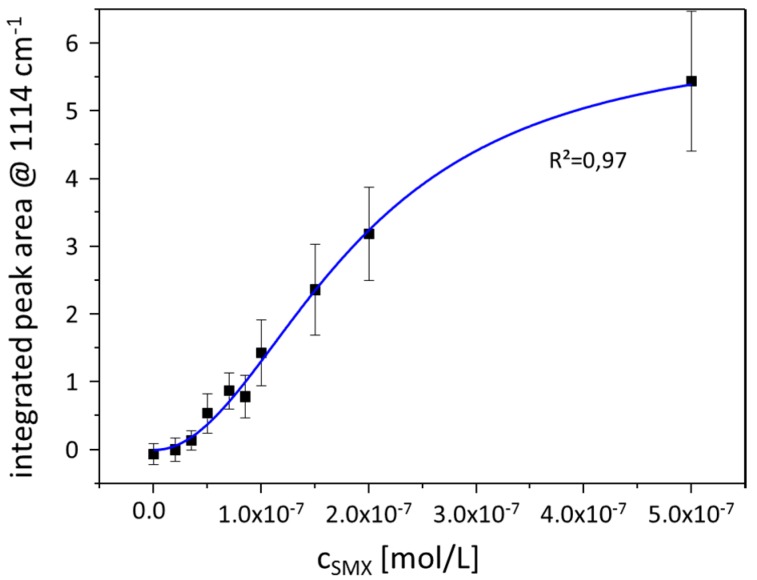
Correlation between the normalized peak area of the marker band at 1114 cm^−1^ and the SMX concentration fitted with a Langmuir–Freundlich profile. The concentration values are identical with the values provided in Figure 3.

Within Figure 5, the peak area change over the entire analyzed concentration range is depicted. The signal evolution reflects the typical behavior known from SERS experiments. Here, the peak area of the SMX marker mode is normalized on the peak area of the silicon marker mode at 521 cm^−1^. The peak area value increases with the concentration and the data points can be fitted by a linear function for the lower concentration range (see Figure 4B). For higher concentrations, a saturation effect occurs and the signal intensity does not increase with the concentration to the same extent as that known for low concentrations. Consequently, the profile of the curve provides information about the interaction behavior of the analyte molecules with the metallic surface. Due to the evanescent character of the electromagnetic field on plasmonic active nanostructures and a decay in signal strength with r^−12^ [36], the SERS signal is dominated by contributions from analyte molecules within the first layer. Analyte molecules within the second layer (or further layers) experience less electromagnetic field intensity, which results in a lower signal enhancement. Thus, the overall signal shows a saturation which is associated with the higher distance of the analyte molecules from the metallic surface. Moreover, only analyte molecules within the first layer can undergo a chemical enhancement since a chemisorption of the analyte molecule is mandatory to do so [37]. Thus, the molecules within the first layer experience both electromagnetic and chemical enhancement, whereas the molecules within the second and further layers will profit only from the electromagnetic enhancement mechanism. Therefore, at low surface coverage, the SERS signal intensity profile follows a Langmuir isotherm [38], describing a monolayer of analyte molecules on the metallic surface. With increasing surface coverage, the SERS signal is not only caused by SMX molecules from the first layer but also from multilayers, in which the SERS signal intensity increases to a lesser extent than for a monolayer coverage. Consequently, the SERS signal intensity follows a Freundlich isotherm for multilayer surface coverage. The correlation between SERS signal intensity and SMX concentration can be described by a combined Langmuir–Freundlich or Sips’ isotherm which supports the conclusion that the contribution to the SERS signal originates from the first as well as from the multilayer coverage of the nanoparticle with analyte molecules. The fitting curve in Figure 5 correlates with the Langmuir–Freundlich isotherm (with the fit parameters *Q_sat_* = 6.14, *K* = 5.33 × 10^13^ and *n* = 2.04) [39]

(1) $$q=Q_{sat}\frac{K\cdot c^{n}}{1+K\cdot c^{n}},$$

which is a combination of the Langmuir isotherm

(2) $$q=\frac{K\cdot c}{1+K\cdot c}$$

and the Freundlich isotherm [38]

(3) $$q=K\cdot c^{\frac{1}{n}}.$$

Therefore, it is concluded that surface coverage with SMX molecules takes place first as a monolayer. Moreover, an additional signal contribution from multilayered SMX molecules is observed based on the signal intensity profile depicted in Figure 5. Nevertheless, there is a certain concentration range around the reversal point of the Langmuir–Freundlich fit, where the peak area of the marker band correlates linearly with the concentration. For the detection of SMX using the TopUp@silicon substrate, this linear range occurs between the SMX concentrations of 2 × 10^−8^ and 2 × 10^−7^ M. Therefore, quantification within this concentration range is achieved.

## 3. Conclusions

The implantation of an internal standard was introduced by using TopUp nanostructures as SERS substrates. Here, the support material is silicon, which results in both the SERS fingerprint information of the analyte molecules and the Raman signal of silicon recorded simultaneously in one spectrum. Although the applied SERS substrates provide background information due to the residues from the fabrication via a bottom-up fabrication process, the analyte molecule sulfamethoxazole is detected within the relevant, sub-µM concentration range. Moreover, it is demonstrated that the linearity between 2 × 10^−8^ and 2 × 10^−7^ M is improved by normalizing the data points on the peak area of the 521 cm^−1^ Raman mode of silicon. Thus, the quantification potential is improved by the proposed normalization procedure. Finally, considering higher concentrations, the signal intensity evolution is fitted by a Langmuir–Freundlich isotherm which reflects the interaction behavior of the analyte molecules with the metallic surface, i.e., forming first a monolayer and, when all binding sites in the first layer are occupied, forming further layers, resulting in less SERS signal enhancement due to the evanescent character of the electromagnetic field generated on plasmonic nanostructures.

## 4. Materials and Methods 

### 4.1. Chemicals

Sulfamethoxazole, hydroquinone, and silver acetate were purchased from Sigma-Aldrich (St. Louis, MI, USA) in the highest available purity. All resists and chemicals mentioned for the fabrication of the gold template structure were purchased from Allresist GmbH (Berlin, Germany).

### 4.2. Gold Template Structure

The gold template structures were fabricated on waferscale by means of electron beam lithography, thermal evaporation, and lift-off techniques. As substrates, 4″ Si wafers are used. First, a two-layer e-beam resist system was spun onto the precleaned wafer. Such two-layer resists are commonly used for lift-off processes and consist of a 70 nm thick ARP617.03-resist on the bottom and a 150 nm ARP6200.09-resist on top. The ARP617 was tempered for 10 min at 200 °C on a hotplate, and the ARP6200 for 3 min at 150 °C. After spin-coating, the resist surface was additionally covered with a 10 nm gold film to avoid charging during the e-beam exposure. The e-beam exposure was performed using a Vistec SB350OS e-beam writer (50 keV). This shaped beam system is equipped with the unique Character Projection technique which allowed the fast quasi-parallel exposure of nanopatterns on large areas [40,41,42]. 

After the beam exposure, the resist was developed for 60 s in AR600-546 and 60 s in IBK/IPA = 1:3, rinsed in IPA (isopropanol), and dry spun afterwards. As an adhesion promoter, a layer of 3 nm Ti was evaporated, followed by 50 nm of gold on top of the resist pattern. The lift-off process was done in an AR600-71 bath for 12 h and the gold island template patterns were created. At the end, an oxygen plasma was applied to remove residual resist and to clean the surface.

### 4.3. Self-Organized Silver Structure

The preparation protocol for the self-organized silver deposition on the gold template structure is described elsewhere [25]. Briefly, the gold template structure was incubated in a mixture consisting of 2 mM hydroquinone in citric buffer (pH 3.8) solution and an aqueous 2 mM silver acetate solution in the ratio 1:2. After 10 min the reduction of silver ions to crystalline silver was stopped by washing the substrates with high purity water. Finally, the as-prepared substrates were dried with compressed air. The created nanostructured surfaces are referred to as TopUp@silicon substrates, since they combine top-down electron beam lithography with bottom-up fabrication of silver nanostructures on silicon wafers.

### 4.4. Instrumentation

The scanning electron microscope (SEM) images were recorded by using a JEOL JSM-6700F microscope (JEOL (Germany) GmbH, Freising, Germany). The samples were investigated without any covering of the nanostructured surface.

The Raman and SERS measurements were performed using a confocal Raman microscope (alpha300, WITec, Ulm, Germany) equipped with a laser line at 488 nm. The Raman spectra of silicon were collected with a 100× objective (NA 0.8) and a laser power of 25 mW. The SERS signals were collected with a 10× objective (NA 0.25) and the laser power after the objective was 3 mW. To avoid sample burning, the measurements were carried out under wet conditions by incubating the substrate in the appropriate analyte solution with a layer of 0.4 cm of the analyte solution above the substrate. The SERS measurements were performed by recording 10 scans for each concentration with the following parameters: 2 points per line, 50 lines per scan, 60 × 60 µm^2^, and integration time of 0.5 s per spot. Thus, it is guaranteed to average over a large area to avoid SERS signal variations due to locally different enhancement properties. 

### 4.5. Data Analysis

The data analysis was performed by applying in-house-developed algorithms using the program language R. The spectra were background corrected by a SNIP (Sensitive Nonlinear Iterative Peak clipping) algorithm [43]. In order to estimate the peak area, the Simpson rule was utilized as implemented in the function *sintegral* from the package *Bolstad* [44].

## 5. Patents

The fabrication of the TopUp SERS substrate is registered within the patent application DE201510004114 (Sophie Patze, Uwe Hübner, Dana Cialla-May).
